# Reading, decrypting, and actuating with light in soft-robotic materials

**DOI:** 10.1038/s41377-026-02383-6

**Published:** 2026-06-26

**Authors:** Sieun Jeon, Dong Ki Yoon

**Affiliations:** 1 Department of Chemistry, Korea Advanced Institute of Science and Technology (KAIST), Daejeon, Republic of Korea; 2 GIST InnoCORE AI-Nano Convergence Initiative for Early Detection of Neurodegenerative Diseases, Gwangju Institute of Science and Technology (GIST), Gwangju, Republic of Korea

**Keywords:** Microresonators, Liquid crystals

## Abstract

An optically interactive soft robotic system based on liquid crystal networks and silk fibroin integrates holographic command storage, encryption, visual display, and actuation into a single soft-matter platform. This approach moves soft robots beyond conventional stimulus–response systems toward architectures that embed information processing directly within the material.

Soft robotics has advanced through compliant material systems^1^ that enable continuous deformation and increasingly adaptive, bio-inspired functions^2^. Liquid-crystalline polymer networks and elastomers have emerged as a particularly compelling strategy^3^, combining mechanical softness with light responsiveness^4,5^, structurally encoded shape change^6^, and multifunctional architectures^7^. The field is now moving toward platforms expected to contribute to sensing^8,9^, information handling^10^, and programmable actuation^3,7,11,12^—yet most systems still rely on a functional separation in which the material body executes movement while command generation and decision-making remain externally assigned. Recent efforts toward physical intelligence^13^, encoded function^14^, and integrated soft sensing^8,9^ point to a more unified view of soft machines, one that more closely couples mechanical response, stimulus selectivity, and information-related functions within the material system itself. The present work exemplifies this direction, showing how information storage, conditional display, and physical execution can be brought into closer correspondence within a single soft-material platform.

In work reported recently in Light: Science & Applications, Zhang and co-workers addressed this challenge by developing an optically interactive soft robotic system that integrates optical information processing directly into the robot’s material architecture^15^. The system encodes task commands as phase holograms in liquid crystal network films, which are reconstructed under specific polarization conditions and presented visually. The same material platform also deforms mechanically in response to light and humidity. The advance lies not in a single function but in integrating command storage, encryption, optical display, and physical execution into a single system (Fig. 1). This represents a step toward coupling information and actuation at the material level.

**Fig. 1:**
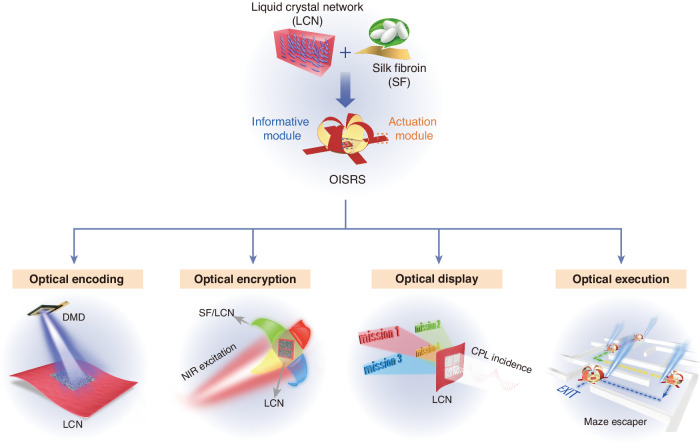
Schematic illustration showing the design of soft robotic system with integrated information encoding, encryption, display, and execution functionalities

The materials strategy is central to this concept. Liquid crystal networks provide photothermal actuation through a splay-aligned structure^3^ in which light-induced disorder drives reversible bending^4^. The same films can also store solid-state phase holograms via controlled photoalignment, enabling direct integration with soft-robotic architectures. Silk fibroin complements this by introducing humidity responsiveness. Above the humidity-induced glass transition threshold, disruption of hydrogen bonds and chain rearrangement allow irreversible shape morphing. This moisture-driven plasticity compensates for the limited thermoplastic behavior of liquid crystal networks and enables the formation of actuators with complex three-dimensional geometries. Together, the bilayer system achieves functions that neither component alone can provide.

An additional layer of encoding is implemented using silk fibroin films doped with upconversion nanoparticles. Under near-infrared excitation, these materials emit distinct fluorescence signals that serve as optical labels. When combined with holographic projection, they enable a hierarchical decoding scheme in which commands are accessible only under defined optical conditions and sequences. With four distinguishable fluorescence channels assigned to four holographic elements, the system supports multiple permutations, only one of which yields the correct instruction. This approach introduces a unique form of material-level information security^16^ for soft robotics.

These principles are demonstrated through two representative systems. A four-armed soft gripper, preconfigured through humidity-induced shaping, performs object sorting guided by holographically encoded instructions and actuated by light. A maze-navigating walker integrates a humidity-responsive structure, a holographic disk, and fluorescence-coded elements into a multi-step decoding architecture. In this system, structural opening, holographic readout, and fluorescence identification must occur in sequence before the correct navigation path is revealed. The resulting behavior depends not only on external stimuli but also on the correct interpretation of layered optical information.

Although the system does not yet achieve full autonomy, it establishes an important framework for coupling optical information with mechanical response in soft materials. Interpretation between optical readout and mechanical execution still relies on external input, and the operating wavelengths are not immediately compatible with direct biological deployment. Even so, the integration of information storage, conditional display, and actuation within a single soft-material platform represents a meaningful conceptual advance. With further progress in sensing and automated decoding, similar strategies may find use in microrobotic systems, implantable devices, and programmable materials. More broadly, this work highlights that future soft robotic systems may benefit not only from advances in actuation but also from more integrated approaches to handling information within the material itself.
